# A114 IMPROVING THE QUALITY OF PARACENTESIS PRACTICES IN PEOPLE WITH ADVANCED CIRRHOSIS IN AN AMBULATORY CARE SETTING

**DOI:** 10.1093/jcag/gwad061.114

**Published:** 2024-02-14

**Authors:** M Sedarous, E Lee, A Verma, K Quinn, G Hirschfield

**Affiliations:** University of Toronto, Toronto, ON, Canada; University of Toronto, Toronto, ON, Canada; University of Toronto, Toronto, ON, Canada; University of Toronto, Toronto, ON, Canada; University of Toronto, Toronto, ON, Canada

## Abstract

**Background:**

Spontaneous bacterial peritonitis (SBP) is a lethal complication of decompensated cirrhosis carrying a 90% mortality rate when left untreated. Despite AASLD recommendations, there is a practice gap regarding fluid cell count (FCC) collection during paracentesis.

**Aims:**

We initiated prospective quality improvement (QI) project to improve FCC collection in an ambulatory care setting from a baseline of 78% to a goal of 100% compliance between September 11, 2022 and April 17, 2023.

**Methods:**

We examined the effects of a quality improvement initiative in 233 adult patient encounters with cirrhosis undergoing paracentesis in an ambulatory setting located within a quaternary care centre in Toronto, Canada within the study timeframe. Using a multidisciplinary approach, the quality improvement initiatives included focused groups, education regarding AASLD guidelines, development of a paracentesis bundle, and streamlining workflow process. Descriptive statistics were collected. Statistical analysis using run charts and p-charts were conducted using QI Macros. Approval was obtained from the QI Review Committee at the institution where this QI initiative took place.

**Results:**

Baseline adherence for collecting ascitic FCC per paracentesis procedure was 78%. Inconsistencies during process mapping such as variations in documentation, printing FCC labels and consistency in FCC collection were identified as root causes. Thus, bedside paracentesis bundles were introduced to standardize process and improve workflow. Post-intervention, the FCC rate increased marginally to 79.6% with no special cause variation identified (Figure 1). Subgroup analysis demonstrated FCC collection rates of 100% amongst registered nurse (RN) and nurse practitioners (NP), 97% amongst resident physicians, and 92% amongst attending physicians. Subsequently, another subgroup analysis was completed demonstrating 97% FCC collection rate amongst general hepatology group, while 40% FCC rate amongst specialty hepatology group. Overall rate of SBP was 1.62% (N=3).

**Conclusions:**

This prospective QI study revealed a gap in guidance pertaining to the role of ascitic fluid cell count collection in patients with cirrhosis undergoing paracentesis in ambulatory settings. Further studies ascertaining rationale for not sending FCC may be helpful in elucidating why there is a gap in clinical practice. Additionally conducting studies in ambulatory settings to assess FCC collection's impact on morbidity and mortality will provide vital evidence and clarity for the role of FCC in this vulnerable patient group.

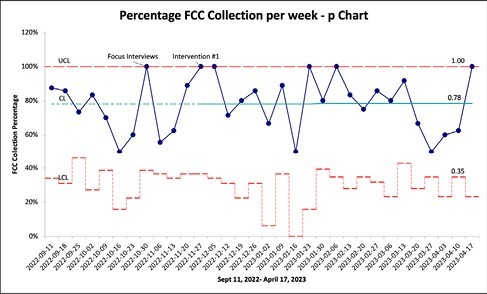

**Figure 1**: P-chart demonstrating percentage FCC Collection per week from September 11, 2022 to April 17, 2023. UCL indicates upper control limit; CL, center line; LCL, lower Control Limit.

**Funding Agencies:**

Toronto Centre for Liver Disease funded the MSc QI program for Mary Sedarous (writer) who completed this study as her MSc QI project.

